# The feasibility of genome-scale biological network inference using Graphics Processing Units

**DOI:** 10.1186/s13015-017-0100-5

**Published:** 2017-03-20

**Authors:** Raghuram Thiagarajan, Amir Alavi, Jagdeep T. Podichetty, Jason N. Bazil, Daniel A. Beard

**Affiliations:** 1 Pratt & Miller Engineering, WK Smith Drive, New Hudson, MI USA; 2 Department of Molecular and Integrative Physiology, University of Michigan, North Campus Research Complex, Ann Arbor, MI USA; 3 Department of Physiology, Michigan State University, 567 Wilson Road, East Lansing, MI USA; 4 Computational Biology Department, School of Computer Science, Carnegie Mellon University, 5000 Forbes Ave., Pittsburgh, PA USA

**Keywords:** Network inference, Reverse engineering, Genetic regulatory networks, GPU

## Abstract

Systems research spanning fields from biology to finance involves the identification of models to represent the underpinnings of complex systems. Formal approaches for data-driven identification of network interactions include statistical inference-based approaches and methods to identify dynamical systems models that are capable of fitting multivariate data. Availability of large data sets and so-called ‘big data’ applications in biology present great opportunities as well as major challenges for systems identification/reverse engineering applications. For example, both inverse identification and forward simulations of genome-scale gene regulatory network models pose compute-intensive problems. This issue is addressed here by combining the processing power of Graphics Processing Units (GPUs) and a parallel reverse engineering algorithm for inference of regulatory networks. It is shown that, given an appropriate data set, information on genome-scale networks (systems of 1000 or more state variables) can be inferred using a reverse-engineering algorithm in a matter of days on a small-scale modern GPU cluster.

## Background

One of the outstanding challenges of systems biology is to reconstruct and simulate genome-scale regulatory networks based on genome-scale data. This challenge is made difficult by the sparseness and noisiness of genome-scale expression and proteomics data [1, 2], as well as the inherent computational complexity of the problem [3, 4]. Specifically, here we consider an ill-posed problem of identifying network models from high-throughput time-course data from large scale dynamical systems [5] using parallel computing platforms.

Graphics Processing Units (GPUs) facilitate parallel calculations in applications that can be scaled from a desktop to a high-performance computing environment. GPU computing has impacted computational modeling with applications in various fields of research such as bioinformatics [6], molecular dynamics [7], and density functional theory [8]. The availability of GPUs has enabled researchers to address larger scale problems and drastically reduce the time taken by simulations compared to traditional methods [6–8].

Inference of network models from high-dimensional data has two bottlenecks: the size of the problem [9], and robustness of the network inference algorithm [10, 11]. The processing power of GPUs can help overcome the first bottleneck by using an algorithm optimized for the parallel architecture of GPUs. To help overcome the second bottleneck, a variety of network inference algorithms have been developed  [10–16]. Bazil et al. developed a method for reverse engineering of genetic pathways based on a distributed parallel algorithm [4, 17]. With this algorithm the number of calculations for a network size of *N* variables scales as $\mathcal {O}(N^2)$ [4]. This $\mathcal {O}(N^2)$ scaling is an improvement over deterministic model-inference techniques, where computation costs scale as $\mathcal {O}(N^3)$, and information theory based approaches, where computational costs scale as $\mathcal {O}(N^2 \log N)$. Even though its computational cost scales as $\mathcal {O}(N^2)$, current applications of this algorithm have been limited to 100–200 variables to obtain results in reasonable timescales [17]. However, the algorithm is perfectly suited for a GPU platform and significant speedups on a GPU platform relative to a central processing unit (CPU) platform are expected. Indeed, adaptation of this algorithm to a GPU platform is expected to facilitate genome-scale network analysis in tractable timescales.

Yet GPU computing is challenging due to the required programming [18]. Effective GPU applications must minimize the data transfer between the host (CPU) and the device (GPU), and the memory requirements on the device (GPU). Implementing the Bazil et al. algorithm on a GPU platform requires consideration of these details to obtain effective performance. A tailored approach is essential to utilize processing power of GPU architecture [19]. Optimum usage of shared memory and asynchronous data transfers [20], exploiting global memory usage and multiple streams of execution [21], and usage of GPU-optimized data structures [22] have been shown to yield significant speedups [19].

This article reports an updated version of the Bazil et al. algorithm tailored for a GPU architecture applied to reverse-engineer a 1000 node network from high-throughput time-course in silico data. The application is developed using the NVIDIA^®^ CUDA^®^ compiler. It centers on solving non-linear ordinary differential equations (ODE) describing the interactions between genes. Two variants of the algorithm utilizing different ODE solvers are implemented on the GPU platform. The first one is ODEINT [23], which utilizes THRUST libraries [24] to solve the ODE on a GPU, and the second one is a LSODA implementation [25] on a GPU [26]. Application to a 1000-variable *in silico* data set illustrates the viability of reverse engineering of genome-scale biological networks. Results from the GPU method were compared with a widely used method called TIGRESS  [15]. TIGRESS combines least angle regression (LARS)  [27] with stability selection  [28, 29] and was ranked among the top five network inference methods in the DREAM5 gene reconstruction challenge  [11].

## Methods

### Distributed network inference algorithm

The distributed algorithm developed by Bazil et al. is suited for analyzing large-scale data sets including, but not limited to, time-course mRNA expression data. The time-course inverse problem of determining regulatory networks is decomposed into *N* one-dimensional problems, one for each of the *N* variables in the network. The algorithm involves searching for maximally likely versions of the one-dimensional model for each state variable. In practice, its application requires integrating millions of different realizations of the basic underlying ODE model.

The governing ODE used to describe regulatory pathways between genes in a network is similar to the ODE described in the community-wide challenge within the DREAM project [10]. The mRNA expression level $x_j$ for the *j*th gene is governed by a mass balance equation [4]:

1 $$\begin{aligned} \frac{{\text {d}}{x}_{j} (t)}{{\text {d}}{t}} = r_j (t) - d_j x_j (t) \text { ,} \end{aligned}$$

with initial condition $x_j (0) = x_{0j}$. The rate at which the *j*th gene is transcribed is $r_j (t)$ and $d_j$ is the degradation constant for the *j*th gene. The rate is modeled by competitive binding of activating and inhibiting transcription factors subject to co-operativity and saturation:

2 $$\begin{aligned} r_j (t) = r_{0,j}\frac{\left( {\sum _{i\in I_{Aj}} \frac{x_i(t-\tau )}{K_{Ai,j}}}\right) ^{n}+e_j}{1+\left( {\sum _{i\in I_{Ij}} \frac{x_i(t-\tau )}{K_{Ii,j}}}\right) ^{n}+\left( {\sum _{i\in I_{Aj}} \frac{x_i(t-\tau )}{K_{Ai,j}}}\right) ^{n}+e_j} \text { ,} \end{aligned}$$

where $I_{Aj}$ and $I_{Ij}$ are sets of indices of variables that act as activators and inhibitors of mRNA level for the *j*th gene. The maximal rate of mRNA production is $r_{0,j}$. The parameter $\tau$ represents a time delay for mRNA transcription, translation, and post-translational signaling events. Cooperative, non-linear binding is assumed with the Hill coefficient $n > 1$ for the binding constants $K_{Ai,j}$ and $K_{Ii,j}$. Externally stimulated or constitutive transcription is captured by the term $e_j$.

According to these governing equations, gene transcription is determined by a competition among inhibitory and activating factors. There is no single weight associated with a given activating or inhibiting edge. Rather each activation interaction has an associated value of $K_{Ai,j}$. When the effector gene concentration/activity is greater than the $K_{Ai,j}$ for a given edge, it has a strong activating effect on the target gene. Similarly, for an inhibitory edge, when the effector gene concentration/activity is greater than the $K_{Ii,j}$ for a given edge, it has a strong inhibitor effect on the target gene.

Since the algorithm developed by Bazil et al. splits the network problem for *N* genes into *N* independent one-dimensional problems (sub-networks) for each gene, these independent one-dimensional problems can be run in parallel to search for trial models for each gene. This implementation of independent sub-networks is described in pseudo-code in Algorithm 1. The ODE solver is run on the GPU so that millions of independent candidate sub-networks can be evaluated simultaneously. A final global network is then generated by combining all the sub-networks generated for each gene and filtering out unlikely interactions. To test the application of GPU computing to this problem, computational costs for simulating large numbers of realizations of the gene expression model are determined for implementations on CPU versus GPU.

The algorithm is implemented using two different ODE solver packages, ODEINT and cuLsoda, on the GPU. ODEINT is developed in a C++ environment by Ahnert and Mulansky [23]. cuLsoda is a CUDA^®^ version of the LSODE ODE solver developed by Hindmarsh and Petzold [30]. To test the performance of these two ODE solvers we use a single-variable model with two activators and no inhibitors. The governing equation (1) reduces to

3 $$\begin{aligned} \frac{{\text {d}}{x}_{1} (t)}{{\text {d}}{t}}&= r_{1} (t) - d_{1} x_{1} (t) \text { ,} \end{aligned}$$

4 $$\begin{aligned} r_{1} (t)&= r_{0,1}\frac{{\left( \frac{x_{2}(t-1)}{K_{A2,1}}+\frac{x_{3}(t-1)}{K_{A3,1}}\right) }^{2}+e_{1}}{1+{\left( \frac{x_{2}(t-1)}{K_{A2,1}}+\frac{x_{3}(t-1)}{K_{A3,1}}\right) }^{2}+e_{1}} \text { ,} \end{aligned}$$

where $x_2$ and $x_3$ represent activators for the target gene 1. Values of $x_{2}(t)$ and $x_{3}(t)$ used in calculating the right-hand side in Eq. (3) are determined using a cubic spline interpolation of time-course pseudo-data for these variables. A value of 1 (arbitrary time units) is used for the time delay $\tau$ and the value of Hill coefficient is set as 2. The binding constants, $r_{0,1}$, $e_{1}$, and $d_{1}$ are varied over a range with minimum values of $10^{-2}$ and maximum values of $10^{2}$.

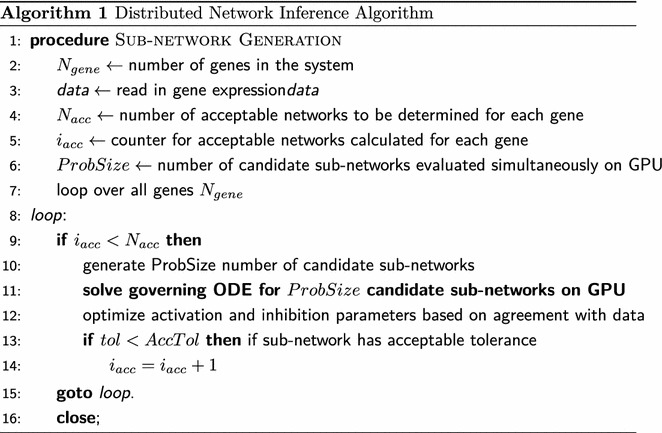



### ODE solvers on the GPU

#### ODEINT

ODEINT uses Thrust [24], which is a parallel algorithms library resembling the C++ Standard Template Library (STL), to run the ODE solver on the GPU. Using Thrust has the advantage that the same application can be run on a multi-core CPU using OpenMP or a GPU by a switch in the compilation instructions. The Dormand-Price 5 algorithm [23] is used to solve the governing ODE presented in Eq. (1), which is an explicit solver not ideal for stiff systems. In general, this is a disadvantage since the governing ODE can be stiff in nature. Here for benchmarking the forward ODE solvers, parameters of the governing ODE in Eq. (3) are fixed so that the problem solved using ODEINT remains non-stiff.

CPU calculations for the case of ODEINT are conducted using a quad-core Intel i5 processor where OpenMP, via Thrust, is used to utilize all the four cores of the CPU. The parameters of the governing ODE are declared with single (using single precision floating point) precision in one case and double precision in another.

#### LSODA

LSODE was originally developed in FORTRAN as part of a systematized collection of ODE solvers by Hindmarsh [25]. Thompson converted a FORTRAN version of LSODA, a variant version of LSODE solver, into a C version to be run on a CPU as well as a CUDA^®^ compatible version to be run on a GPU [26]. A full Jacobian is calculated by LSODA requiring a number of extra calls to the right hand side of the governing ODE presented in Eq. (1). LSODA switches automatically between stiff and non-stiff methods so the user does not need to choose the appropriate method. An Adams method [25] is used for non-stiff problems and the backward differentiation formula 5 (BDF5) method [25] is used for stiff problems. Stiff solvers require more memory to record previous time steps and matrices used for solving implicit equations. The Thompson version of LSODA is used by Zhou et al. in the CUDA-sim package written in Python [31]. Zhou et al. report a speedup of around 50 fold, on a single Tesla C2050 GPU relative to a single Intel Core i7 Extreme Edition CPU, for a single non-stiff ODE which is solved for a variety of different parameters. For comparisons using LSODA speedup is determined on a Tesla K20 GPU relative to a single core of an Intel Xeon E5 Sandy Bridge processor. Another SODA implementation in CUDA is cupSODA which relies on a C version of LSODA. cupSODA is a cross-platform tool which can be run on multiple operating systems. Speedup relative to COmplex PAthways SImulator (COPASI [32]) was found to be in the range of 23× to 86× for different test models [33]. The GPU used for the test models is a NVIDIA GeForce GTX 590 compared with a quad-core CPU Intel Core i7- 2600.

The LSODA/CUDA implementation of sub-network identification component of the Bazil et al. algorithm is distributed on GitHub [34].

### Network identification

The GPU implementation of the reverse-engineering algorithm is validated by applying it to identify connections for a target gene in a 10 gene network generated in silico. This allows us to verify whether the GPU implementation of the algorithm performs equivalently for the same test problems used by Bazil et al. [4]. Performance of the GPU implementation is then analyzed for a 1000-node network, which is also generated in silico.

#### 10-Gene network

The 10-gene network model illustrated in Figure 3 of Bazil et al. [4] is reverse-engineered first to get an initial benchmark and as a test of functionality of the algorithm on a GPU.

#### 1000-Gene network

To test speed and accuracy of the reverse-engineering algorithm a 1000 gene in silico network is generated. The method is implemented using MATLAB R2013b (The Mathworks, Inc.). The network generation algorithm generates connections for each gene and assigns kinetic parameters associated with these connections described in Eq. (1).

**Fig. 1:**
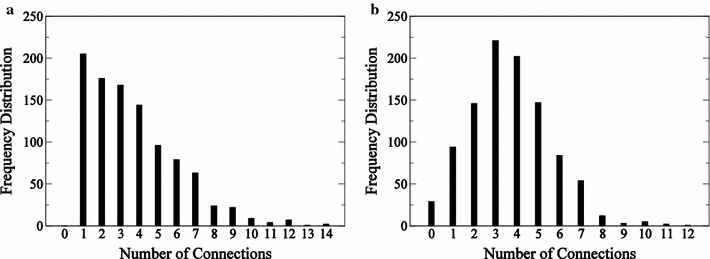
1000-node network characteristics. Histogram of incoming edges (**a**) and outgoing edges (**b**) are presented for the 1000-gene network

The number of connections to each gene are determined from a random distribution in order achieve a power law decay in the number of incoming edges into each gene. Figure 1 illustrates frequency distributions of the edges coming into and going out of the genes in the network. The initial condition for each gene is set as 1 at time $t=0$. The binding constants and other parameters of the governing ODE of Eq. (1) are bounded. The binding constants $K_\text {A}$ and $K_\text {I}$ are bounded between 0.25 and 9.75. The values of the $r_0$ and *d* are randomly generated for each target gene and $e_j$ is bounded between a value of 1 and 21. The time delay for mRNA transcription, translation, and post-translational signaling events represented by $\tau$ is set as one hour. A delay differential function is used to generate time-course profiles of all the genes using MATLAB. The data is generated for 12 time points (over 11 h). The time points are equally spaced and range from 0 to 11.

The two independent experiments generated in silico are done by stimulating different subsets of genes for each experiment. External stimuli are associated to a certain subset of genes which are chosen at random. In the case of experiment 1, 250 of them are activated externally, whereas in the case of experiment 2, 200 of them are activated externally.

### Comparison to TIGRESS

The TIGRESS MATLAB code was downloaded from the SVN repository hosted by researchers at MINES ParisTech [35]. The 1000 Node Network in-silico data was converted into TIGRESS input data format. A scores matrix was obtained with predictions of connections among the genes and transcription factors. The percentage recovery (number of true edges recovered by TIGRESS in %) and false positive rate per gene were calculated for 4 threshold values. Here threshold value is the minimum value above which a true edge is recovered. The threshold or cutoff metric used in TIGRESS is user controlled. To infer the regulatory network from expression data, a score is computed to assess the evidence that each candidate regulation is true. Then a true regulation pair is predicted for which the score is larger than threshold. TIGRESS only focuses on finding a good ranking for the candidate regulation, by reducing score so as to push the true regulation to the top of the list. This method lets the user control the level of false positive and false negative that is acceptable by the user.

TIGRESS was ranked in the top 3 GRN inference methods at the 2010 DREAM5 challenge. It performs on par with other state of the art such as GENIE3 method for in silico network [36]. However, it does not perform as well as GENIE3 in in vivo networks. This is perhaps due to the fact the assumed linear relationship used between Transcription Factor and Target Gene used by TIGRESS is an oversimplification.

## Results and discussion

### Computation costs for ODE solvers: CPU versus GPU

#### ODEINT

**Fig. 2:**
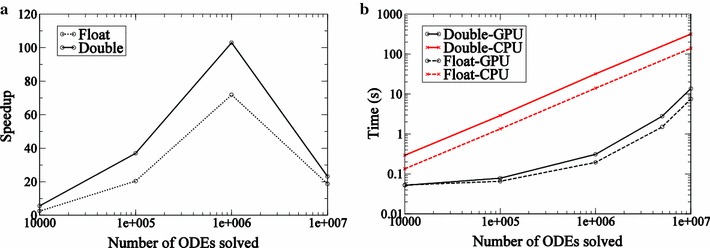
Performance statistics for ODEINT. Speedup (**a**) and time statistics (**b**) for solving ODEs obtained on a Tesla K20 GPU relative to time taken on 8 cores of Intel Xeon E5 Sandy Bridge processor using ODEINT. These statistics are generated for the test problem described in Eq. (3)

Speeds of calculation on the GPU versus the CPU are compared in Fig. 2. A speedup of greater than an order of magnitude is observed when the number of ODEs solved on the GPU exceeds a million. (Speedup is defined as the time to complete integrations of a given number of realizations of the model on the CPU divided by the time taken on the GPU). State variables declared as single precision led to $\approx$2× speedup relative to the case where state variables are declared as double precision, when problem sizes are larger than a million.

For the CPU implementation to provide useful speedups, the time spent on a single kernel call must be long compared to the latency to access data from memory. Simulation times are illustrated in Fig. 2b. We can see, from either the speedup plot or the simulation times, that a single kernel call to the GPU has to be approximately the size of a million ODE integration calls or more to achieve an order of magnitude speedup on the GPU relative to a multi-core CPU. One million independent one-dimensional ODEs are integrated in a fraction of a second on the GPU and in seconds on the multi-core CPU.

#### LSODA

**Fig. 3:**
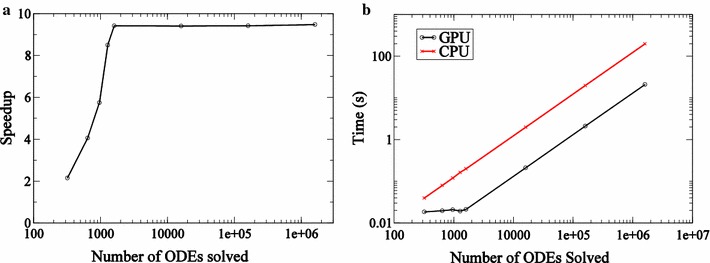
Performance statistics for LSODA. Speedup (**a**) and time statistics (**b**) for solving ODEs obtained on a Tesla K20 GPU relative to time taken on a single core of an Intel Xeon E5 Sandy Bridge processor using LSODA. These statistics are generated for the test problem described in Eq. (3)

Speedup statistics are presented in Fig. 3a. Only double precision designation is considered for state variables. The speedup achieved by LSODA is found to be an order of magnitude for problem sizes larger than approximately $10^3$. Since the stiff solver requires more memory the number of ODEs that can be solved simultaneously on the GPU using LSODA is lower when compared with ODEINT. We find that no more than approximately 2000 jobs may be submitted in a single kernel call. For applications described below, jobs are submitted in blocks of 1600 ODEs iteratively. This number was determined via empirical experimentation. As a result, GPU speedup saturates when the number of ODEs solved on the GPU is greater than 1600. Comparison of timescales for the simulations on the Intel Xeon CPU and Tesla K20 GPU are presented in Fig. 3b. The time taken by the single core of Intel Xeon processor is linear with *M*, the number of ODEs integrated, as expected for a serial process. In the case of the Tesla K20 GPU it becomes linear for problem sizes greater than 1600.

### 10-Gene in silico network

**Fig. 4:**
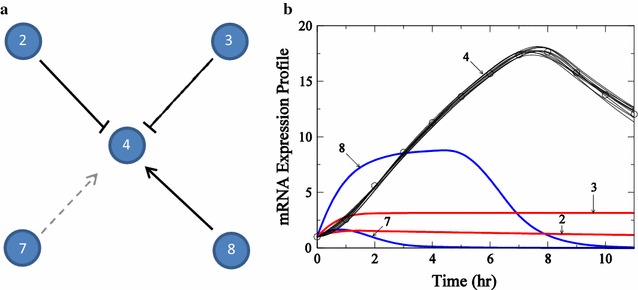
Description of subnetwork for gene #4. Subnetwork topology (**a**) and mRNA expression profiles (**b**) for target gene #4 from the 10-gene network analyzed by Bazil et al. [4]. In **a**, *solid black lines* represent edges recovered by the algorithm, which are present in the true network and *dashed gray line* represents edge not recovered by the algorithm, but which is present in the true network. In **b**, the mRNA expression profile described by the regulator genes determined by the algorithm is presented for the target gene 4. The profiles for the regulator genes are presented as *cubic splines* using mRNA expression data. The number against each profile represents the gene index

Data from the 10-gene network model illustrated in Figure 3 of Bazil et al. [4, 17] were analyzed here to determine putative regulatory interactions associated with one gene in the network. The specific gene analyzed is gene ID #4 in the network, the same variable and same data used by Bazil et al. to test the first step in the inference algorithm. This step, which represents a major bottleneck in the process, involves finding a large number of “subnetworks” for a given variable in the network, and then determining which regulatory interactions (or ‘edges’ in the network) are required to explain the data. Both the “True”, and inferred regulatory networks associated with gene ID #4 in this example and time-course data for this variable are shown in Fig. 4a.

The true network associated with target gene ID #4 has 2 activators (genes 7 and 8) and two inhibitors (genes 2 and 3). The algorithm works by finding as many models (sets of activators and inhibitors) that can explain the time course data as possible. In practice, several thousand candidate subnetworks are generated at random. (In the GPU implementation 1600 trial networks are evaluated in a single call to the GPU). These subnetworks are evaluated by finding optimal parameter values (for activation and inhibition constants associated with each model) that best fit the time course data. Trial subnetworks that can effectively fit the data are accepted as potential connections. These recovered connections are described for gene ID #4. Figure 4b shows simulated time courses for 10 models which are determined to effectively fit the data. Finally, the relative frequency with which a given edge appears in the set of putative subnetworks is taken as a measure of the likelihood of that edge belonging to the true network underlying the dynamical system that generated the data.

**Fig. 5:**
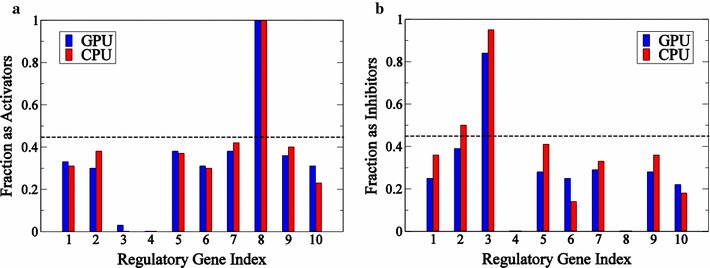
Histograms of activators and inhibitors for gene #4. Value of fractions that each gene appears as an activator (**a**) or an inhibitor (**b**) in the ensemble of subnetworks generated for time course illustrated in Fig. 4

Histograms of inferred activator and inhibitor edges for this system are illustrated in Fig. 5. These histograms indicate the relative frequency that each edge appeared in a total of 2051 trial networks for the GPU and 500 trial networks for the CPU are found to effectively match the time-course data for this variable. Using the value of 0.45 (as determined by Bazil et al.) as an appropriate cutoff to select edges, our results are similar to those of Bazil et al. [4]. Activation of gene ID #4 by gene ID #8 is found in all sampled subnetworks, and inhibition by gene 3 is found in greater than 80% of sampled subnetworks. These two true positives were recovered by Bazil et al. as well. In addition, in the current implementation the edge associated with inhibition by gene ID #2 is present in approximately 50% of sampled subnetworks.

Although this test problem represents an idealized data set, the important finding is that the GPU implementation of the algorithm returns effectively the same results as the CPU implementation. This is an independent validation for the GPU version of the algorithm. On the architectures used here, the GPU implementation is approximately 6 times faster than the CPU implementation. This speedup is obtained by comparing time taken to generate 2051 trial networks on the GPU and 500 trial networks on the CPU. If same number of networks (2000) on both CPU and GPU are generated then the speedup factor on the GPU reaches approximately 24.

**Fig. 6:**
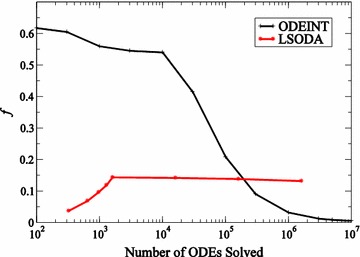
Resource usage on the GPU. Fraction of time in a simulation spent in data transfer (*f*) between host (CPU) and device (GPU) for ODEINT and LSODA GPU ODE solvers

For any application, obtaining useful speedup on a GPU architecture requires reducing the amount of time spent in data transfer between the host (CPU) and the device (GPU). For NVIDIA^®^ devices the NVIDIA^®^ profiler gives insight into how the two ODE solvers, ODEINT and LSODA, perform on the GPU. The fraction of total simulation time spent on data transfer between host and device, represented by *f*, relative to the size of the problem *M*, the number of ODEs solved, is illustrated in Fig. 6. In the case of ODEINT, *f* drops down considerably as the problem size goes up yielding larger speedups for problem sizes larger than a million. This is why the number of jobs must be close to a million for the ODEINT solver in order to obtain any significant speedup on the GPU. In the case of LSODA, *f* does not dominate to the same extent for smaller problem sizes and saturates after the problem size exceeds 1600.

More than an order of magnitude speedup is observed, close to 100$$\times$$ in some cases, for the Tesla K20 GPU relative to an Intel Xeon E5 8 cores processor using ODE solver ODEINT. An order of magnitude speedup is observed for the GPU relative to a single core of Intel Xeon E5 8 cores processor utilizing ODE solver LSODA. This is comparable to speedups found in other studies done on the GPU [31, 37]. The cost of a Tesla K20 GPU is approximately $2000 (February 2017), which is about an order of magnitude larger than the cost of a single quad core CPU. Thus, based only on the costs of the processing units, the GPU and CPU deliver comparable per-dollar performance. NVIDIA^®^ has developed CUDA as well as GPU hardware since release of Tesla K20 GPUs. However, there are other avenues for speedup which can be explored such as: Shared memory and asynchronous data transfers [20], exploiting global memory usage and multiple streams of execution [21], and usage of GPU-optimized data structures [22]. However, factoring in the cost of the computer chassis, and power consumption, the economic advantage of the GPU becomes apparent. Taking into account the ease of programming on the CPU, with relatively less attention paid to memory handling with respect to GPUs, it is evident that developing the application on a CPU environment is relatively easier. Hence, GPU computing as an alternative for large scale biochemical network simulations has to be considered on a case-by-case basis with consideration to factors such as programming effort, timing for simulations, power consumption, and cost of the machines.

### 1000-Gene in silico network

**Fig. 7:**
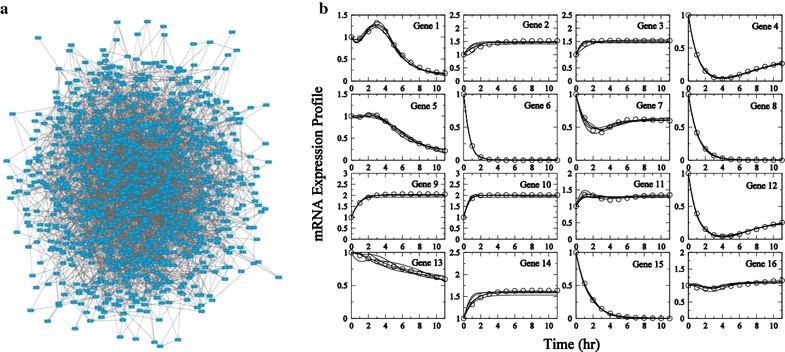
Description of the 1000-node network. Network representation of the 1000 node in silico network is presented in **a**. mRNA expression profiles for the first 16 genes for Experiment 1 are presented in **b**. In silico data are plotted as *circles*. Subnetwork model fits to data are plotted as *solid lines*. Visualization of the network uses Cytoscape [38]

To determine the performance characteristics of the algorithm applied in a realistic setting, the inference algorithm is applied to time-course in silico data generated in silico from a 1000 gene network. Figure 7a illustrates the connections for the 1000 gene network, with 3700 connections or close to 4 connections per gene. Determining the true 3700 actual connections out of the possible $2 \times 10^6$ is an extremely ill-posed problem.

We generated time-course in silico data from this network from two independent simulation experiments. This approach is used to represent two independent experiments on a biological system. For these two experiments, the internal network connections and kinetic parameters remain unchanged. Different dynamic behavior in the two experiments is obtained through two different sets of external stimuli, as detailed in the appendix. In brief, a random subset of the 1000 genes is activated in each experiment by assigning a non-zero value for the terms $e_j$ in Eq. (1).

Expression profiles of first 16 genes are illustrated in Fig. 7b for Experiment 1. Many profiles are similar in nature, with repeated motifs include simple exponential decay. Genes 2, 3, 9, and 14 show the trend of expression levels saturating after the initial time points. Since the algorithm developed here is purely data driven and it is not able to distinguish between activation or inhibition effects of genes with similar profiles, yielding a large number of false positives. As will be illustrated below, performing multiple independent experiments provides an effective means of reducing the number of false positives.

The time taken to reverse-engineer connections for this 1000 node network for one experiment is 13.1 days on 5 T K20 GPUs. Here the regulatory network determined for two genes, gene ID #645 and gene ID #614, are analyzed in greater detail. Gene 645 is a case where our strategy succeeds and gene 614 is a case where the algorithm is less successful.

**Fig. 8:**
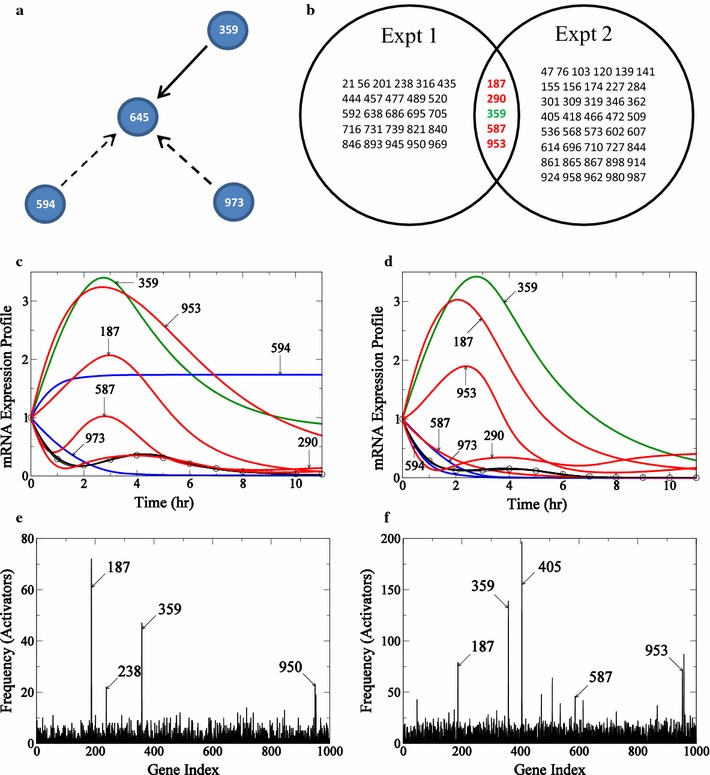
Determination of incoming edges for gene #645 in 1000-node network. The true connections for gene 645 is presented in **a**. Connections recovered by the algorithm are shown in *solid black lines* and those not recovered are shown by *dashed lines*. In **b** the intersection of activators from reverse-engineering networks from the two experiments is presented in the form of a Venn diagram. In **c** and **d**, connections in the actual network recovered in each experiment are shown in *green*, and the false positives common to both experiments are shown in *red*. mRNA expression profiles for gene 645 for the two experiments with profiles from Experiment 1 are presented in **c** and profiles from Experiment 2 are presented in **d**. The profile for gene 645 is represented by *circles*, true edges recovered by the algorithm are shown in *green*, activators appearing as false positives are shown in *red*, and connections missed by the algorithm are shown in *blue*. **e** (Experiment 1) and **f** (Experiment 2): histogram of frequency of the number of times an activator appears in all the acceptable “submodels” generated by the algorithm for target gene 645 is presented

The “true” network for gene 645 consists of three activators 359, 594, and 973 and is illustrated in Fig. 8a. Applying our algorithm to determine subnetworks for this gene that can fit the data from each individual experiment, $10^7$ trial models were evaluated, with at least 5000 subnetworks able to fit the in silico data. Assembling the edges that appear with significant frequency in the sets of acceptable subnetworks we find 31 putative activator genes based on analysis from Experiment 1 and 46 putative activator genes from the analysis of Experiment 2. The 31 putative activators from Experiment 1 and 46 putative activators from Experiment 2 are chosen from the ensemble of all subnetworks as the activators that appear with a frequency of a $p_\text {value}$ of $<0.01$ relative to random selection.

The frequency distribution of putative activators for this gene is presented for the two experiments in Fig. 8e and f. Each of these sets of putative activators, identified based on $p_\text {value}$ of $<0.01$ relative to random selection, is listed in Fig. 8b. There is one true positive gene 359 identified from both experiments. The mRNA expression profiles of relevant genes from the two experiments are shown in Fig. 8c and d, respectively. In summary, for this gene, our algorithm identifies one true positive edge. The intersection of the putative activators from the two independent experiments include this true positive as well as four false positives. Taking the intersection of sets of identified activators, we dramatically reduce the number of false positives.

**Fig. 9:**
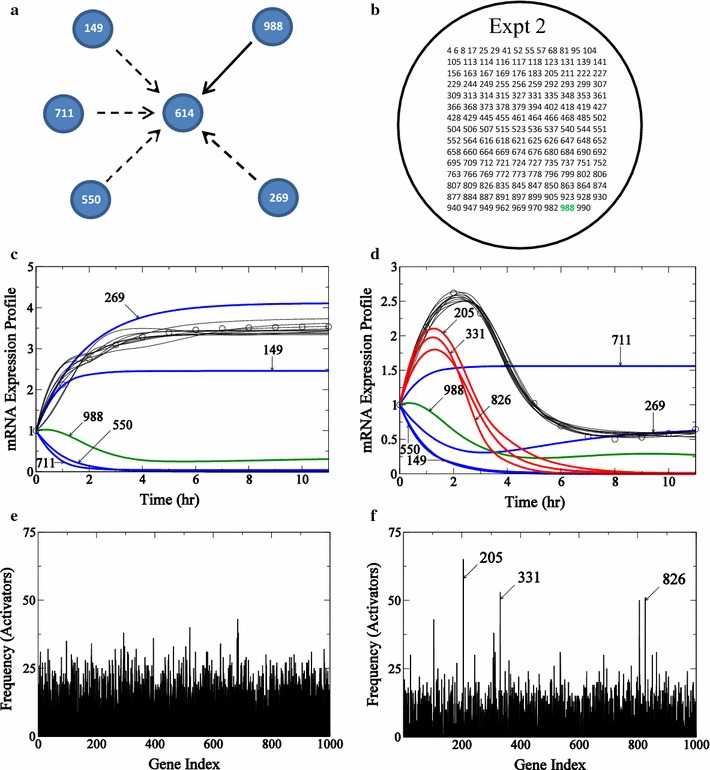
Determination of incoming edges for gene #614 in 1000-node network. The true connections for gene 614 is presented in **a**. Connections recovered by the algorithm are shown in *solid black lines* and those not recovered are shown by *dashed lines*. In **b** only results from the accepted experiment are shown in *right circle* and the results reject from Experiment 1 are show in *circle with dashed lines*. Connections in the actual network recovered in Experiment 2 are shown in *green*. In **c** and **d**, connections in the actual network recovered in each experiment are shown in *green*, and the false positives common to both experiments are shown in *red*. mRNA expression profiles for gene 614 for the two experiments with profiles from Experiment 1 are presented in **c** and profiles from Experiment 2 are presented in **d**. The profile for gene 614 is represented by *circles*, true edges recovered by the algorithm are shown in *green*, activators appearing as false positives are shown in *red*, and connections missed by the algorithm are shown in *blue*. **e** (Experiment 1) and **f** (Experiment 2): histogram of frequency of the number of times an activator appears in all the acceptable “submodels” generated by the algorithm for target gene 614 is presented

Results for gene 614 are illustrated in Fig. 9. Here the predicted expression time course for Experiment 1 is a simple monotonic saturating increase. The frequency distribution of identified activators for this experiment is shown in Fig. 9c. Here, in contrast to the frequency distributions for gene 645, there are no clear outliers from Experiment 1 for gene 614. This is because the time-course profile for the target gene contains little information to select from the trial models. Experiment 2 for this gene does contain useful information, and there are 153 gene activators identified as potentially significant, as illustrated in Fig. 9b. For this case, although the false positive rate is high, 1 of the 5 true positives appear in the set of significant edges obtained from Experiment 2. The expression time courses for the top three activators (gene 205, gene 331 and gene 826) for Experiment 2 appearing in the frequency distribution in Fig. 9f are shown in Fig. 9d.

For this case, since we obtain no useful information from Experiment 1, it is not useful to take the intersection of identified edges to improve the false positive rate. For the 1000 gene in silico data set analyzed here, $58.5\%$ of genes fall into the category of gene 645, with both experiments yielding sets of positive edges. For $22.0\%$ of genes, only one of the two experiments yields useful information. For roughly $19.5\%$, neither experiment yields significant putative edges.

**Fig. 10:**
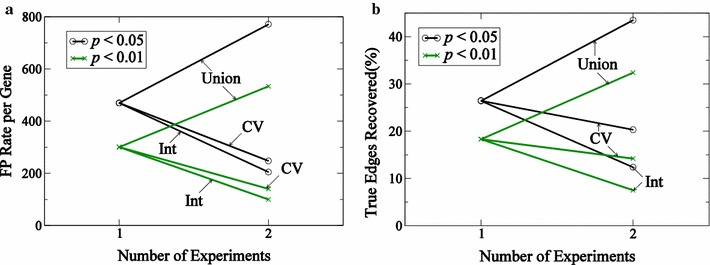
Performance statistics for the reverse-engineering algorithm. False positive rate and fraction (**a**) of true edges recovered (**b**, in %) for set of 1000 genes analyzed using a sensitivity analysis are presented. Results using two cutoff values, 0.01 and 0.05, using a $p_\text {value}$ relative to a chance that a connection appears using a random binomial distribution. Union represents a union of sets of results obtained from both experiments, *CV* represents the cutoff metric based upon co-efficient variation mentioned in the text, and *Int* represents intersection of results obtained from both experiments

Figure 10 reports the performance of the algorithm, using two different values for $p_\text {value}$ are used to select significant putative regulating genes (activators and inhibitors). For a single experiment, $18.3\%$ of true edges are recovered using $p_\text {value} = 0.01$ and $26.4\%$ using $p_\text {value} = 0.05$. As we have observed, the number of false positives is high, and is higher for high values of $p_\text {value}$ used. Taking the union of identified genes from the two independent experiments increases the fraction of true edges recovered, but also increases the false positive rate. Taking the intersection leads to reduction in the false positive rate by a factor of 5, but also leads to reduction in true edge recovery rate which is $7.5\%$ for $p_\text {value}=0.01$. To improve the edge recovery rate, we analyze which experiments have useful information using a co-efficient of variation ($CV>0.05$) metric, which is standard deviation divided by the mean. This metric is used to determine whether the algorithm can make a prediction depending on the time-course profile of the target gene. Based on this criterion, the information obtained from a given experiment for a given target gene is accepted or rejected before taking the intersection of results from the two independent experiments. If one of the two experiments is judged to be uninformative for a given target gene, the intersection of results from two experiments is not applied. Applying this step improves the edge recovery rate from 7.5 to $14.2\%$ for $p_\text {value} = 0.01$ as illustrated in Fig. 10b. This increase in edge recovery rate is accompanied by increase in number of false positives.

### Comparison to TIGRESS

In TIGRESS, the percentage true edge recovered was higher at lower threshold values with the highest recovery rate of $6.2\%$ for no threshold used, $1.9\%$ for a threshold value of 0.01 and $0.3\%$ for a threshold value of 0.1. The performance of TIGRESS on this problem is summarized in Table 1. The true edge recovery rates attained with TIGRESS are significantly lower than true edge recovery rate from our reverse engineering method where the true edges recovered are $7.2\%$, for one experiment, and $14.2\%$, for two experiments. This $14.2\%$ recovery rate is associated with a 140 false positive per gene rate. Thus the GPU method performed better in recovering true edges with a higher false positive rate. The cutoff metric or threshold value is determined by user for TIGRESS.

**Table 1:** TIGRESS performance described in terms of true edges recovered and false positive rate

Threshold	True edges recovered (%)	FP rate per gene
0	6.2	98.5
0.01	1.9	21.1
0.1	0.3	4.1
0.2	0.1	1.9

Thus our algorithm performed better than TIGRESS for this trial problem in recovering true edges, while generating more false positives. TIGRESS generated significantly fewer edges overall and a marginally lower false positive rate. Moreover, this comparison is biased in favor of our algorithm since the in silico data used for the test are generated from a model that uses the same underlying mathematical structure as assumed by our inference algorithm. Practical applications to real data may draw on multiple algorithms, each with its own relative advantages and disadvantages  [10–16].

## Conclusions

The algorithm presented here is able to reverse-engineer network connections in a $1000\text {-node}$ network from high dimensional time-course data within 2 weeks on 5 T K20 GPUs. The parallel algorithm scales linearly with number of processors employed.

By leveraging multiple GPU’s, network inference on the scale of thousands of variables becomes feasible. Thus, genome-scale biological network inference is becoming feasible using the current generation of GPU-architecture computing clusters. The network prediction of GPU method is comparable to other GRN methods such a TIGRESS. The main advantage of GPU method is in its ability to process large data sets in a relatively short period of time.

The data-driven approach used by the algorithm depends on the information available for each node for a particular experiment. So in order to identify a given interaction in the network, data must contain extractable information pertaining to a particular interaction. Furthermore, when multiple genes show similar time-course behavior, it is difficult to distinguish true interactions from false positives. This shortcoming can potentially be overcome by utilizing robust clustering algorithms when applying the reverse-engineering algorithm to high dimension biological time-course data [17].
